# Glymphatic system and mild traumatic brain injury: a mini review

**DOI:** 10.3389/fnins.2025.1705690

**Published:** 2025-10-16

**Authors:** Pihla Miettinen, Begüm Utz, Ivette Bañuelos-Cabrera, Eugene Golanov, Ziv Lenzner, Leonardo Lara-Valderrábano, Lasse Välimaa, Adrian Harel

**Affiliations:** ^1^Medicortex Finland Oyj, Turku, Finland; ^2^Department of Neurosurgery, Houston Methodist Hospital, Houston, TX, United States; ^3^Bezalel Academy of Arts and Design, Jerusalem, Israel

**Keywords:** aquaporin 4 (AQP-4) water channels, concussion, enlarged perivascular spaces (ePVS), glymphatic system, sleep, diffusion tensor imaging along the perivascular spaces (DTI-ALPS), mild traumatic brain injury (mTBI)

## Abstract

Since the discovery of the glymphatic system in 2012, research on this brain-wide fluid exchange pathway has focused on understanding its role in different neurological diseases. Mild traumatic brain injury (mTBI) is a prevalent, yet often undiagnosed, condition that increases the risk of developing debilitating neurodegenerative diseases. mTBI may lead to impaired glymphatic system function and, therefore, accumulation of metabolic waste in the brain. In this review, we summarize 24 studies (10 rodent, 13 human, 1 both) published during 2013–2025, reporting post-mTBI changes in the glymphatic system. According to pre-clinical models, potential post-mTBI drivers of glymphatic dysfunction include depolarization of aquaporin 4 water channels and sleep deprivation. In studies on humans, evidence is contradictory; some studies show reduced post-mTBI glymphatic activity, while others report increased activity. However, these studies used different patient populations, which were likely exposed to different mTBI types and post-injury time frames. Furthermore, studies on humans used non-invasive imaging techniques, which only indirectly measure glymphatic activity. Taken together, these inconsistencies point to major gaps in the field, highlighting the need for standardized injury classification and post-injury time frames, and more direct measurements of glymphatic activity in humans. Notably, sleep deprivation, post-concussive symptoms, and cognitive impairment have often been linked to post-injury glymphatic dysfunction. Nevertheless, to better understand mTBI implications on glymphatic system functioning, further research is needed. Such research could help develop novel diagnostics or treatment strategies for mTBI and potentially mitigate the long-term risks of developing neurodegenerative disorders.

## 1 Introduction

The glymphatic system is a brain-wide fluid transport pathway discovered in 2012 (Iliff et al., 2012). This pathway facilitates the exchange of fluid and solutes between cerebrospinal fluid (CSF) and interstitial fluid (ISF), eventually draining into the meningeal and cervical lymphatic system (Bohr et al., 2022). The glymphatic system is crucial in clearing waste from the brain, but it is also involved in the delivery of nutrients and active substances to the brain (Astara et al., 2023; Ding et al., 2023).

**Figure 1:**
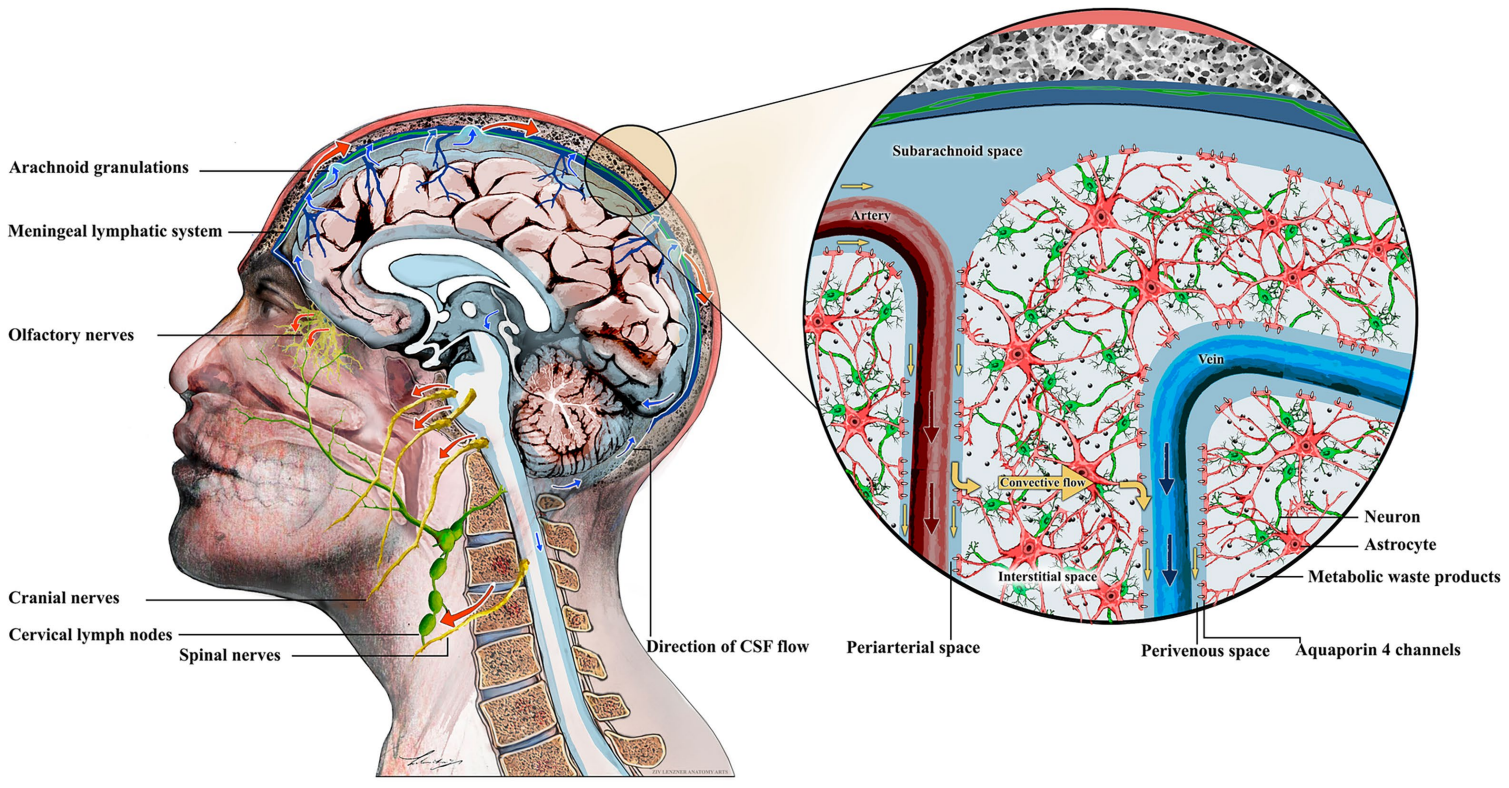
The glymphatic system in the brain. From the subarachnoid space, cerebrospinal fluid (CSF) enters periarterial spaces along penetrating arteries and further into brain parenchyma through aquaporin-4 (AQP-4) channels, which are highly expressed in astrocytic end-feet surrounding brain vasculature. In the brain parenchyma, CSF mixes with interstitial fluid (ISF). This mixture, carrying metabolic waste, flows through the brain parenchyma due to convection. Eventually, this fluid drains from the interstitium into perivenous spaces along cerebral veins. From there, it exits through pathways along olfactory nerves, cranial and spinal nerves, meningeal lymphatic vessels, and arachnoid granulations. This fluid ultimately drains to cervical lymph nodes (CLNs) and reenters circulation via subclavian veins.

In the glymphatic pathway, a combination of forces, such as arterial pulsation and respiration, drives CSF influx from the subarachnoid space into periarterial spaces surrounding penetrating arteries (Figure 1) (Ng Kee Kwong et al., 2020; Bohr et al., 2022). These periarterial spaces are lined with astrocytic end-feet that highly express aquaporin 4 (AQP-4) water channels, which facilitate CSF entry into the interstitial space (Ng Kee Kwong et al., 2020). In the brain interstitium, through the combined effects of diffusion and advection, CSF mixes with ISF (Bohr et al., 2022). This mixture of fluids, carrying solutes, drains from the interstitium into perivenous spaces along cerebral veins (Ng Kee Kwong et al., 2020; Bohr et al., 2022). From there, it exits via pathways along olfactory nerves, cranial and spinal nerves, meningeal lymphatic vessels, and arachnoid granulations (Rasmussen et al., 2018; Ding et al., 2023). Ultimately, this fluid drains to cervical lymph nodes (CLNs), and through subclavian veins, returns to general circulation (Bohr et al., 2022; Ding et al., 2023).

The glymphatic system is 90% more active during sleep, suggesting state-dependent clearance of metabolic waste products from the brain (Christensen et al., 2020). Sleep is associated with a 60% increase in interstitial space in the brain, leading to elevated CSF-ISF exchange, and consequently, improved clearance of *β*-amyloid from the brain (Xie et al., 2013). Thus, sleep disturbances related to aging and various diseases can lead to glymphatic dysfunction and accumulation of metabolic waste (Astara et al., 2023). In fact, dysfunction of the glymphatic system has been implicated in several conditions, including sleep deprivation, aging, Alzheimer’s disease (AD), and traumatic brain injury (TBI).

TBI is a global health problem, affecting nearly 70 million people each year (Dewan et al., 2019). Waste clearance failure may contribute to TBI pathophysiology, which involves, following cellular damage due to an impact to the head, release of harmful molecules to the brain interstitium (Kvist et al., 2023; Butler et al., 2024). Current diagnostics lack sensitivity to detect mild TBI (mTBI, also known as concussion), which constitutes over 90% of all TBI cases (Maas et al., 2022). Undetected mTBI increases the risk of repetitive brain injuries which, in turn, may lead to neurodegenerative diseases, such as AD or chronic traumatic encephalopathy (CTE) (McKee et al., 2023).

In this mini review, we summarize the literature on the role of the glymphatic system in mTBI. Targeting glymphatic function may provide a new mTBI diagnostic or treatment method.

## 2 Pre-clinical mTBI models: impaired glymphatic flow

In rodent models of TBI, glymphatic pathway dysfunction is evidenced by a 60% reduction in perivascular CSF influx (Iliff et al., 2014). Using contrast-enhanced magnetic resonance imaging (CE-MRI), one study demonstrated mTBI-induced dysfunction in both CSF influx and efflux (Li et al., 2020). Glymphatic dysfunction in post-injury rodents emerges as early as 2 h, with the effect persisting at least up to 1 month (Iliff et al., 2014; Bolte et al., 2020).

Rodent models have also served to investigate potential mechanisms underlying post-TBI glymphatic dysfunction. In mTBI and moderate TBI models, the perivascular polarization of AQP-4 is disrupted at the end-feet of reactive astrocytes (Ren et al., 2013; Eisenbaum et al., 2024). Furthermore, the genetic knock-out of AQP-4 aggravates post-TBI glymphatic dysfunction, leading to an accumulation of tau protein, S100β, glial fibrillary acidic protein, and neuron-specific enolase. This aggregation of proteins seems to promote, in the post-injury brain, the development of neurofibrillary pathology, neuroinflammation, and neurodegeneration (Iliff et al., 2014; Plog et al., 2015).

Similar to diminished AQP-4 polarization, post-TBI sleep deprivation can reduce glymphatic flow (Plog et al., 2015). In a murine model of repetitive blast mTBI, researchers observed chronic impairment of glymphatic function as well as emergence of sleep–wake disruption. The observed sleep disturbances resembled daytime sleepiness and nighttime insomnia typical in post-TBI humans (Braun et al., 2024).

Some findings point towards region-specific post-mTBI changes in glymphatic activity. For example, one study reported, in their repetitive mTBI model, increased glymphatic influx but decreased efflux. In addition, these changes were region-specific, only affecting limbic structures and the olfactory bulb (Christensen et al., 2020). Another study also highlighted region-specific differences in glymphatic flow 24 h after repetitive mTBI. Using near-infrared (NIR) imaging, those researchers observed significantly slower flow to the anterior regions and faster flow to the posterior regions of the brain. Interestingly, AQP-4 expression was decreased in regions of higher NIR dye intensity, suggesting that CSF accumulates in the perivascular spaces, leading to increased perivascular volume. Moreover, they found reduced glymphatic efflux to the CLNs, indicating impaired waste clearance in the brain and blood accumulation at the CLNs, implying the role of the glymphatic system in removing mTBI-related extravascular blood (Michalaki et al., 2025). While Michalaki et al. (2025) proposed that reduced AQP-4 expression and polarization in posterior regions may lead to CSF accumulation and impaired clearance, Christensen et al. (2020) did not discuss upstream mechanisms for the region-specific changes observed in the glymphatic activity. Therefore, there is a need for further mechanistic studies to determine why certain brain regions are more vulnerable to changes in glymphatic flow after mTBI.

## 3 Post-mTBI alterations in human glymphatic function

Clinical studies of the glymphatic system and mTBI utilize non-invasive imaging techniques that provide an indirect measure of glymphatic function (Table 1). The most commonly used techniques are structural MRI-based enlarged perivascular spaces (ePVS) and diffusion tensor imaging along perivascular spaces (DTI-ALPS) (Zhuo et al., 2024). The DTI-ALPS technique is discussed in detail by Taoka et al. (2017, 2024), who proposed the method for evaluating human glymphatic system activity. The ePVS technique is reviewed by Ramirez et al. (2016) and Sepehrband et al. (2019). The ePVS evaluates the number and volume of visible PVS in the region of interest on T2-weighted MRI images with or without T1-weighted MRI (Sepehrband et al., 2019; Zhuo et al., 2024). Increased ePVS burden (number and volume) indicates impaired glymphatic function (Yamamoto et al., 2024; Zhuo et al., 2024). The DTI-ALPS index, derived from diffusion tensor imaging (DTI), assesses the diffusivity of the perivascular space along the deep medullary veins at the level of the lateral ventricular bodies (Morita et al., 2023; Zhuo et al., 2024). A lower DTI-ALPS index signifies impaired glymphatic function (Zhuo et al., 2024).

**Table 1:** Clinical studies on the glymphatic function following mTBI.

Study	Population	Injury	Imaging window	Imaging method	Imaging outcome	Glymphatic function	Correlating factors
Braun et al. (2024)	Veterans	Blast mTBI	N/A	ePVS	Increased PVS burden	↓	Poor sleep
Dai et al. (2023)	General	mTBI	N/A	DTI-ALPS	Increased ALPS index	↑	N/A
Garcia et al. (2024)	General	mTBI	7 days of injury	ePVS	Increased PVS number	↓	N/A
Huang et al. (2024)	Young boxers	SRC	N/A	DTI-ALPS	Increased ALPS index	↑	WM damage, CI
Joseph et al. (2024)	Student athletes	mTBI	7 days of injury	ASL	Decreased GCRs; once clinically cleared, increased GCRs	↓	N/A
Jung et al. (2024)	Former football players	RHI	N/A	ePVS	Increased PVS volume	↓	CI
Miyata et al. (2025)	Former professional athletes	Repetitive mTBI	N/A	DTI-ALPS	Decreased ALPS index	↓	CI
Morita et al. (2023)	Older adults who played contact sports in youth	N/A	N/A	DTI-ALPS	Decreased ALPS index	↓	CI
Piantino et al. (2021)	Veterans	mTBI	N/A	ePVS	Increased PVS burden	↓	Poor sleep, PCSs
Powell et al. (2024)	SOF soldiers	mTBI	N/A	ePVS	Increased PVS burden	↓	N/A
Wang et al. (2024)	General	Chronic mild-to-moderate TBI	N/A	DTI-ALPS	Decreased ALPS index	↓	CI
Yamamoto et al. (2024)	Veterans	Blast mTBI	N/A	ePVS	Inflammatory markers correlate with PVS burden	N/A	N/A
Yang et al. (2024)	General	mTBI	1 month of injury	DTI-ALPS	Decreased ALPS index	↓	WM damage, CI
Zhuo et al. (2024)	General	mTBI	14 days & 6–12 months of injury	ePVS and DTI-ALPS	Increased PVS burden at 14 days; increased ALPS index from 14 days to 6–12 months	↓	PCSs, sleep improvement from 14 days to 6–12 months

ALPS, along perivascular space; ASL, arterial spin labeling; CI, cognitive impairment; DTI-ALPS, diffusion tensor imaging along perivascular spaces; ePVS, enlarged perivascular spaces; GCRs, glymphatic clearance rates; mTBI, mild traumatic brain injury; PCSs, post-concussive symptoms; PVS, perivascular space; RHI, repetitive head impact; SOF, special operations forces; SRC, sports-related concussion; WM, white matter.

Notably, many studies on mTBI and the glymphatic system are conducted on military personnel or contact sports athletes. Both groups of individuals are frequently exposed to head impacts, while military personnel are also exposed to blasts that can cause brain injuries. Repetitive head injuries, frequent in these groups, are a major health concern since they increase the risk of developing neurodegenerative diseases, including AD and CTE (McKee et al., 2023).

In studies on U.S. military veterans and soldiers, the PVS burden was positively correlated with the number of mTBIs sustained (Piantino et al., 2021; Braun et al., 2024; Powell et al., 2024). Poor sleep modulated this relationship by increasing the effect of each mTBI on PVS volume (Piantino et al., 2021; Braun et al., 2024). Furthermore, the elevated PVS burden was linked to increased severity of post-concussive symptoms (PCSs) (Piantino et al., 2021). Similarly, in a study with former American football players, exposure to repetitive head impacts was associated with larger PVS volume. Moreover, the increased PVS volume was linked to impaired cognitive and executive functioning (Jung et al., 2024). In one study on PVS burden, between veterans with blast mTBI history and those without a history of brain injuries, no differences emerged. However, that study identified a positive correlation between PVS burden and concentrations of several proinflammatory biomarkers from CSF and plasma in only blast-mTBI patients (Yamamoto et al., 2024).

Some studies have shown that PVS burden is acutely increased following mTBI (7 days after injury) and is linked to an increased number of PCSs (14 days after injury) (Garcia et al., 2024; Zhuo et al., 2024). Conversely, one study found that a history of repetitive head impacts is linked to decreased ePVS number at the acute post-injury stage (Garcia et al., 2024).

Studies that used the DTI-ALPS index as a measure of glymphatic activity have also yielded mixed results. Some have reported lower post-injury DTI-ALPS indices, supporting the hypothesis of glymphatic dysfunction in mTBI (Wang et al., 2024; Yang et al., 2024). In those studies, the lower DTI-ALPS index correlates with cognitive impairment (CI), with white matter damage potentially modulating the effect (Morita et al., 2023; Wang et al., 2024; Yang et al., 2024). In one study, compared to those aged 66 and above, who played non-contact sports in their youth, those that played contact sports exhibited CI and lower DTI-ALPS indices (Morita et al., 2023). The long-lasting effects of contact sports on glymphatic function were further highlighted by a recent study. Therein, former professional athletes with a history of long-term exposure to repetitive mTBI displayed CI with lower DTI-ALPS indices (Miyata et al., 2025).

In contrast, other studies have shown higher post-mTBI DTI-ALPS indices (Dai et al., 2023; Huang et al., 2024). In a retrospective study, both MRI-positive (signs of mTBI) and MRI-negative (no signs of mTBI) patients with mTBI had elevated DTI-ALPS indices, with the elevation being more pronounced at younger ages (Dai et al., 2023). Similarly, compared to healthy individuals engaging in regular exercise, young boxers with sports-related concussions (SRCs) showed higher DTI-ALPS indices. In this subpopulation, a higher DTI-ALPS index correlated with white matter microstructural damage and CI (Huang et al., 2024). A longitudinal study reported that DTI-ALPS indices increase from the acute phase (14 days post-injury) to the chronic phase (6–12 months post-injury), correlating with sleep improvement (Zhuo et al., 2024).

A non-invasive MRI technique, arterial spin labeling (ASL), uses arterial blood water protons as an endogenous contrast agent to enable the evaluation of cerebral perfusion (Joseph, 2021; Kamagata et al., 2024). If images are acquired at long time point intervals, 3D ASL can capture the residual signal from the labeled protons within ISF. This way, 3D ASL can help measure glymphatic activity. Reduced clearance rates of these protons indicate impaired glymphatic outflow (Joseph, 2021). A recent study demonstrated the utility of 3D ASL in measuring human glymphatic activity. In that study, ASL MRI showed reduced glymphatic clearance rates (GCRs) in student athletes within 7 days of mTBI. Furthermore, once those athletes were clinically cleared to return to their sport, GCRs significantly improved (Joseph et al., 2024).

In summary, human studies have reported both increases and decreases in glymphatic function following mTBI. Taken together, these discordant results likely originate from fundamental differences between studies, including patient populations, types and timing of injury, and imaging methodologies. Some authors have proposed mechanistic explanations, suggesting that reduced post-mTBI glymphatic activity may result from loss of AQP-4 polarization, whereas increased activity could represent a compensatory response to facilitate waste removal. These possibilities are examined in greater detail in the following Discussion.

## 4 Discussion

In this review, we presented an overview of preclinical and clinical studies that examined post-mTBI glymphatic system function. These findings have discrepancies, which may result from differences in models, study populations, and methods. While some studies reported an overall decrease in glymphatic influx (Iliff et al., 2014; Li et al., 2020), two studies, which used mTBI models, observed a region-specific increase in glymphatic influx and decrease in efflux (Christensen et al., 2020; Michalaki et al., 2025). These studies argued that their findings of region-specific changes also reflect an overall dysfunction in the glymphatic system (Christensen et al., 2020; Michalaki et al., 2025). One of these studies also found a region-specific decrease in glymphatic influx, further pointing towards glymphatic dysfunction (Michalaki et al., 2025). However, the other study reported no change in influx nor efflux in some regions. Those researchers argued that, since mTBI is associated with a build-up of neurotoxic waste, a clearance rate comparable to that of a healthy animal is insufficient, yet again supporting the theory of glymphatic dysfunction (Christensen et al., 2020).

Clinical studies reporting decreased post-mTBI glymphatic function have suggested that AQP-4 polarization loss observed in mTBI animal models could also occur in humans (Piantino et al., 2021; Jung et al., 2024; Yang et al., 2024). According to this theory, AQP-4 polarization loss can impair CSF transport from the PVS into the interstitial space, leading to chronic PVS dilation. As a result, glymphatic flow becomes disrupted, promoting waste accumulation in the brain parenchyma (Piantino et al., 2021). However, to date, no direct evidence exists of AQP-4 polarization loss in humans. In fact, a recent study examining post-mortem cortical tissues from veterans exposed to blast TBI observed no AQP-4 polarization changes (Braun et al., 2024).

Those studies which found enhanced post-injury glymphatic function have hypothesized that this enhancement may reflect a compensatory mechanism to reduce secondary damage (Dai et al., 2023; Garcia et al., 2024; Huang et al., 2024). Dai et al. (2023) argued that increased glymphatic function in both MRI-positive and MRI-negative patients with mTBI might reflect a compensatory mechanism that, by facilitating metabolic waste clearance, decreases secondary degeneration. Furthermore, this compensatory enhancement of glymphatic function might be related to increased post-injury AQP-4 expression (Dai et al., 2023; Huang et al., 2024). Some mTBI model studies have observed increased AQP-4 expression although its implications for glymphatic function remain elusive (Higashida et al., 2011; Tomura et al., 2011). One study not only found increased AQP-4 expression in their repetitive blast mTBI model but replicated the finding in post-mortem cortical tissues from veterans exposed to blast TBI. However, increased AQP-4 expression was associated with delayed glymphatic dysfunction, contradicting the theory of a compensatory mechanism (Braun et al., 2024).

Contradictory findings in studies on mTBI and glymphatic function may also stem from study population variability. Participants in these studies were from diverse backgrounds, including current and former non-professional or professional athletes, veterans, soldiers, and the general population. Therefore, they are also likely to be exposed to different mTBI types: SRC, blast mTBI, repetitive mTBI, and non-specified mTBI. Each mTBI type may involve unique pathophysiological changes, including those affecting glymphatic function. Moreover, every brain injury is unique, further complicating data interpretation.

Another potential source of variability in studies on post-mTBI glymphatic function is time elapsed from injury to brain imaging. In some studies, imaging was performed relatively acutely, during 1 to 2 weeks after injury, whereas in other studies, months or even years may have passed since injury. In many cases, time elapsed from injury to imaging is unreported, and imaging findings are correlated with a history of a single or multiple mTBI. Furthermore, some studies are retrospective, using imaging data collected in the past.

Importantly, both ePVS and DTI-ALPS are indirect measures of glymphatic function. While intrathecal gadolinium-enhanced MRI remains the gold standard for directly assessing glymphatic activity in humans, its invasiveness limits clinical applicability. Consequently, non-invasive techniques are gaining prominence. Notably, Zhang et al. (2021) found a significant correlation between the DTI-ALPS index and glymphatic clearance as measured with intrathecal gadolinium (Zhang et al., 2021). Novel non-invasive methods, such as ASL MRI, are being developed; ASL offers the advantage of shorter scan times (under 20 min) compared to DTI-ALPS (up to 2 h) (Joseph et al., 2024). For further discussion of emerging imaging techniques, see reviews by Kamagata et al. (2024) and Botta et al. (2025).

Despite conflicting findings on post-mTBI glymphatic function, several correlating factors are consistently reported. One of these factors is sleep deprivation, which worsens post-mTBI glymphatic impairment. Moreover, from the acute to chronic post-injury stage, glymphatic function increases, correlating with sleep improvement (Zhuo et al., 2024). However, mTBI patients frequently experience sleep disruption in acute care settings due to regular neurological assessments and sedative use (Plog et al., 2015). Therefore, to improve glymphatic function and post-mTBI patient outcomes, targeting sleep could provide a treatment strategy (Braun et al., 2024).

Furthermore, post-mTBI glymphatic dysfunction is associated with CI and PCSs. Patients with impaired glymphatic function after injury tend to experience more severe and frequent PCSs. Among these, balance and memory problems are the most prominent (Piantino et al., 2021; Zhuo et al., 2024). Impaired performance in cognitive and executive function tests is often linked to post-mTBI glymphatic dysfunction. This CI may result from white matter damage, which correlates with post-injury glymphatic dysfunction. According to this theory, post-mTBI glymphatic dysfunction leads to protein accumulation, which in turn, results in neuronal damage, ultimately diminishing cognitive function (Huang et al., 2024; Wang et al., 2024; Yang et al., 2024).

## 5 Conclusion

In conclusion, mTBI appears to alter glymphatic system activity; however, findings remain inconsistent, with reports of both increased and decreased activity. These discrepancies likely reflect methodological limitations and variability across studies. To address this, future research should prioritize the standardization of imaging windows and injury classification to enhance comparability across investigations. Additionally, the development of multimodal imaging pipelines may provide more comprehensive insights into glymphatic function post-injury. Establishing longitudinal patient registries and dedicated glymphatic imaging databases will support investigation of glymphatic changes over the course of a mTBI. Advancing our understanding of post-mTBI glymphatic dynamics through such standardized and integrated approaches may inform the development of novel diagnostic tools and therapeutic strategies, ultimately improving patient outcomes.
